# Supplementary material for: Continued value of the serum alpha-fetoprotein test in surveilling at-risk populations for hepatocellular carcinoma
Source: PLoS One. 2020 Aug 26;15(8):e0238078. doi: 10.1371/journal.pone.0238078 (PMC7449471; doi:10.1371/journal.pone.0238078)
Supplement: S1 Fig — (A) overall mortality and (B) cancer-specific mortality of the AFP and AFP+US groups after propensity score matching in Model 1. Both overall and cancer-specific mortalities were significantly different in the two groups (Ps<0.001, respectively, by the log-rank test). (DOCX) [file pone.0238078.s001.docx]

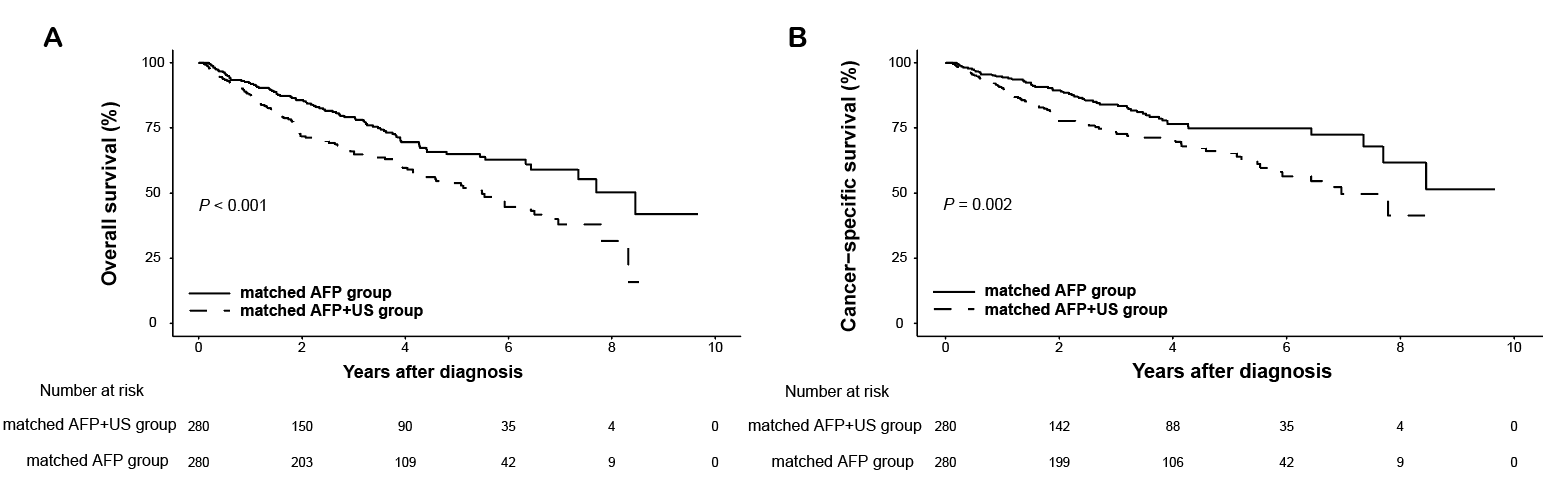


**S1 Fig.** (A) overall mortality and (B) cancer-specific mortality of the AFP and AFP+US groups after propensity score matching in Model 1. Both overall and cancer-specific mortalities were significantly different in the two groups *(Ps*<0.001, respectively, by the log-rank test)
