# Supplementary material for: Continued value of the serum alpha-fetoprotein test in surveilling at-risk populations for hepatocellular carcinoma
Source: PLoS One. 2020 Aug 26;15(8):e0238078. doi: 10.1371/journal.pone.0238078 (PMC7449471; doi:10.1371/journal.pone.0238078)
Supplement: S2 Fig — (A) Overall mortality and (B) cancer-specific mortality of the AFP and AFP+US groups after propensity score matching in Model 2. Neither overall nor cancer-specific mortalities were significantly different in the two groups (P = 0.200 and P = 0.230, respectively, by the log-rank test) (DOCX) [file pone.0238078.s002.docx]

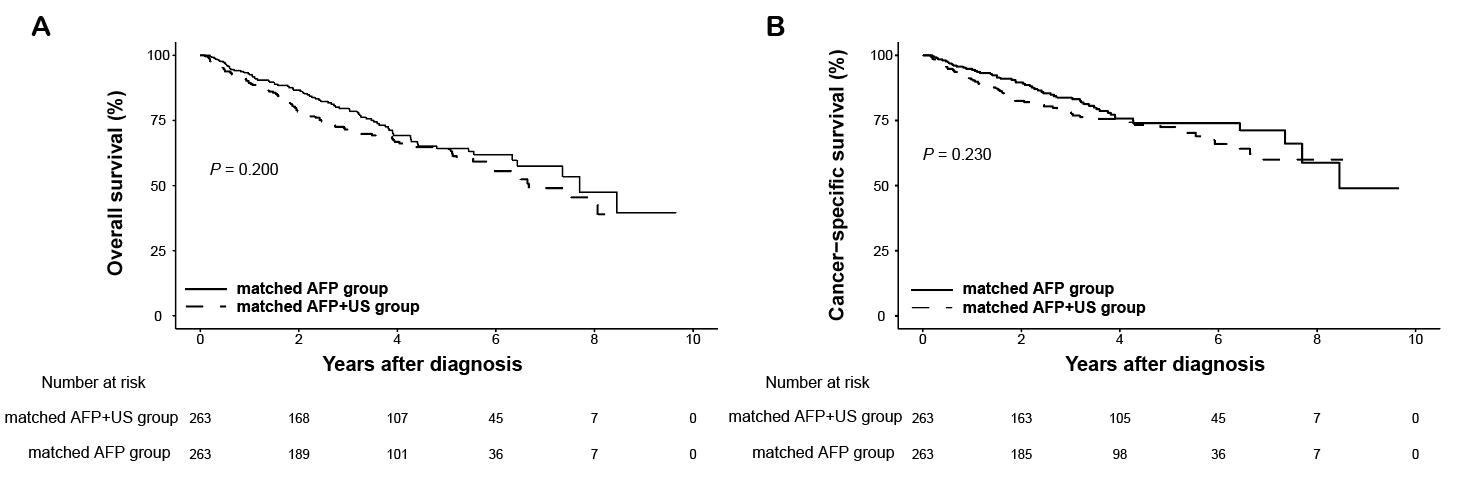


**S2 Fig.** (A) Overall mortality and (B) cancer-specific mortality of the AFP and AFP+US groups after propensity score matching in Model 2. Neither overall nor cancer-specific mortalities were significantly different in the two groups (*P*=0.200 and *P*=0.230, respectively, by the log-rank test).
