# Supplementary material for: Continued value of the serum alpha-fetoprotein test in surveilling at-risk populations for hepatocellular carcinoma
Source: PLoS One. 2020 Aug 26;15(8):e0238078. doi: 10.1371/journal.pone.0238078 (PMC7449471; doi:10.1371/journal.pone.0238078)
Supplement: S3 Fig — The differences between groups with respect to (A) overall mortality, and (B) cancer-specific mortality, were significant after adjusting for the calculated lead time (tumor doubling time 120 days, P<0.001 and P<0.001, respectively by the log-rank tests). (DOCX) [file pone.0238078.s003.docx]

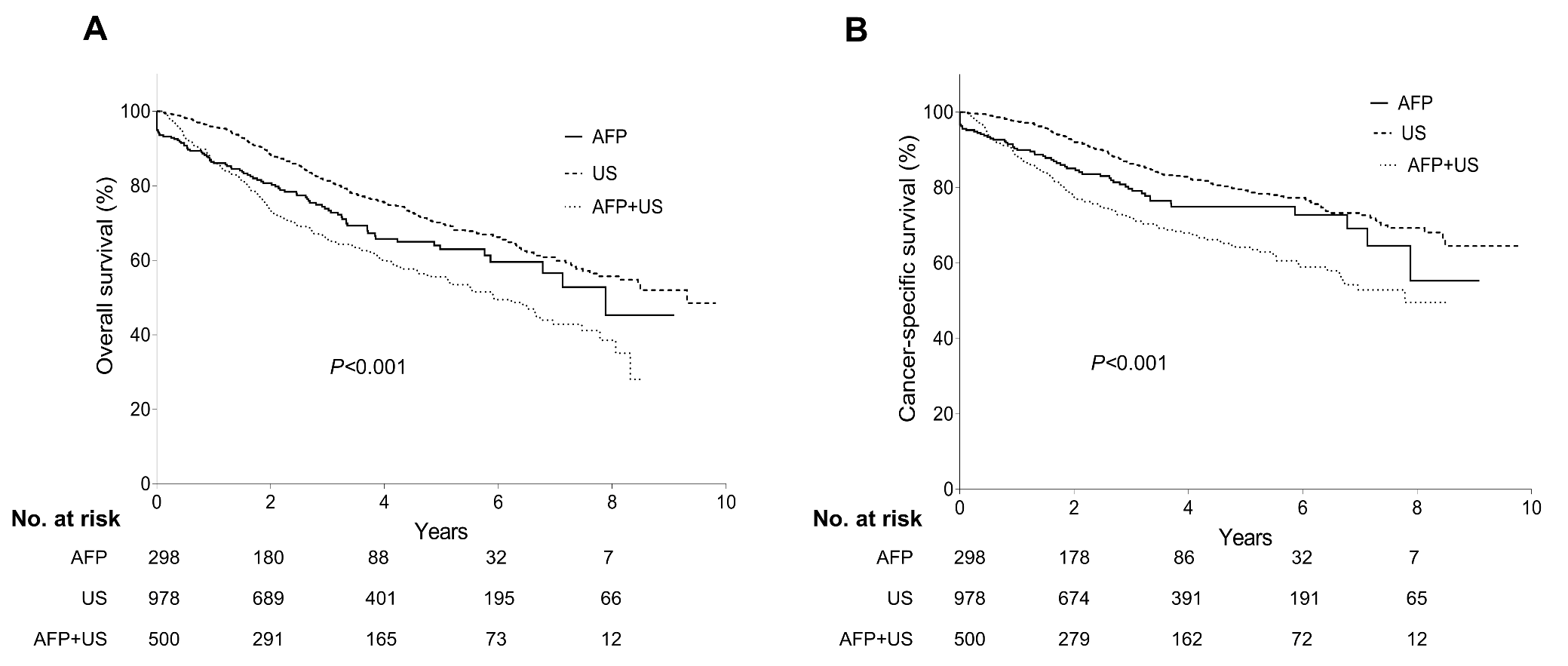


**S3 Fig.** Corrected survival of patients according to abnormal results of the HCC screening tools. The differences between groups with respect to (A) overall mortality, and (B) cancer-specific mortality, were significant after adjusting for the calculated lead time (tumor doubling time 120 days, *P*<0.001 and *P*<0.001, respectively by the log-rank tests).
