# Supplementary material for: Continued value of the serum alpha-fetoprotein test in surveilling at-risk populations for hepatocellular carcinoma
Source: PLoS One. 2020 Aug 26;15(8):e0238078. doi: 10.1371/journal.pone.0238078 (PMC7449471; doi:10.1371/journal.pone.0238078)
Supplement: S1 Table — (DOCX) [file pone.0238078.s005.docx]

**S1 Table.** Independent pre- and post-screening parameters related to survival in the entire HCC cohort (based on the AJCC system)

| **Variable** | **Overall morality** | | | | | | **Cancer-specific mortality** | | | | | |
| --- | --- | --- | --- | --- | --- | --- | --- | --- | --- | --- | --- | --- |
|  | **Model 1** | | | **Model 2*** | | | **Model 1** | | | **Model 2*** | | |
|  | **HR** | **95% CI** | ***P*** | **HR** | **95% CI** | ***P*** | **HR** | **95% CI** | ***P*** | **HR** | **95% CI** | ***P*** |
| Group |  |  |  |  |  |  |  |  |  |  |  |  |
| AFP+US group | 1 |  |  | 1 |  |  | 1 |  |  | 1 |  |  |
| AFP group | 0.60 | 0.47-0.78 | <0.001 | 0.73 | 0.57-0.95 | 0.017 | 0.55 | 0.41-0.75 | <0.001 | 0.68 | 0.50-0.92 | 0.012 |
| US group | 0.53 | 0.43-0.64 | <0.001 | 0.57 | 0.47-0.69 | <0.001 | 0.46 | 0.37-0.58 | <0.001 | 0.49 | 0.39-0.62 | <0.001 |
| Male sex | - | - | - | - | - | - | 1.57 | 1.19-2.07 | 0.001 | 1.52 | 1.15-2.00 | 0.003 |
| Diabetes | 1.21 | 0.98-1.48 | 0.074 | 1.14 | 0.93-1.40 | 0.211 | - | - | - | - | - | - |
| Positive history of alcohol consumption | 1.37 | 1.15-1.63 | 0.001 | 1.31 | 1.10-1.56 | 0.002 | 1.21 | 0.96-1.53 | 0.114 | 1.22 | 0.96-1.54 | 0.103 |
| HBV infection | 0.61 | 0.47-0.78 | <0.001 | 0.58 | 0.48-0.71 | <0.001 | 0.68 | 0.53-0.87 | 0.003 | 0.83 | 0.65-1.08 | 0.161 |
| HCV infection | 1.31 | 0.96-1.81 | 0.094 | 1.18 | 0.85-1.64 | 0.316 | 1.17 | 0.77-1.80 | 0.459 | 1.03 | 0.67-1.60 | 0.890 |
| Liver Cirrhosis | 1.11 | 0.80-1.53 | 0.540 | 0.88 | 0.63-1.22 | 0.435 | - | - | - | - | - | - |
| Ascites | 2.36 | 1.59-3.49 | <0.001 | 2.01 | 1.34-3.04 | 0.001 | 2.61 | 1.58-4.31 | <0.001 | 2.25 | 1.36-3.74 | 0.002 |
| MELD score | 1.08 | 1.05-1.12 | <0.001 | 1.08 | 1.04-1.11 | <0.001 | 1.05 | 1.01-1.10 | 0.010 | 1.03 | 0.98-1.07 | 0.289 |
| Platelet count <100k/mm^3^ | 1.39 | 1.16-1.67 | <0.001 | 1.12 | 0.93-1.35 | 0.245 | 1.19 | 0.94-1.50 | 0.141 | 0.90 | 0.71-1.14 | 0.382 |
| Infiltrative type of HCC | 5.95 | 4.35-8.14 | <0.001 | 2.52 | 1.79-3.55 | <0.001 | 6.83 | 4.88-9.56 | <0.001 | 2.50 | 1.73-3.62 | <0.001 |
| AJCC stage |  |  |  |  |  |  |  |  |  |  |  |  |
| Stage IA |  |  |  | 1 |  |  |  |  |  | 1 |  |  |
| Stage IB |  |  |  | 1.46 | 1.13-1.87 | 0.004 |  |  |  | 2.12 | 1.51-2.97 | <0.001 |
| Stage II |  |  |  | 1.37 | 1.06-1.78 | 0.017 |  |  |  | 1.68 | 1.18-2.39 | 0.004 |
| Stage IIIA |  |  |  | 3.00 | 2.04-4.40 | <0.001 |  |  |  | 4.03 | 2.53-6.41 | <0.001 |
| Stage IIIB |  |  |  | 4.30 | 3.01-6.16 | <0.001 |  |  |  | 7.43 | 4.86-11.36 | <0.001 |
| Stage IV |  |  |  | 6.88 | 4.68-10.10 | <0.001 |  |  |  | 12.91 | 8.24-20.23 | <0.001 |
| Curative treatments |  |  |  | 0.33 | 0.27-0.40 | <0.001 |  |  |  | 0.29 | 0.23-0.37 | <0.001 |

*Adjusted for AJCC stage, receipt of curative treatment, and all variables in Model 1.

HCC, hepatocellular carcinoma; AJCC, American Joint Committee on Cancer; HR, hazard ratio; CI, confidence interval; AFP, alpha-fetoprotein; US, ultrasonography; HBV, hepatitis B virus; HCV, hepatitis C virus; MELD, model for end-stage liver disease.
