# Supplementary material for: Continued value of the serum alpha-fetoprotein test in surveilling at-risk populations for hepatocellular carcinoma
Source: PLoS One. 2020 Aug 26;15(8):e0238078. doi: 10.1371/journal.pone.0238078 (PMC7449471; doi:10.1371/journal.pone.0238078)
Supplement: S2 Table — (DOCX) [file pone.0238078.s006.docx]

**S2 Table. Baseline characteristics of the AFP and AFP+US groups in the unmatched cohort and propensity score-matched cohorts using variables included in Model 1 and Model 2, respectively**

| **Variable** | **Unmatched cohort** | | | | **Matched cohort (Model 1*)** | | | | **Matched cohort (Model 2†)** | | | |
| --- | --- | --- | --- | --- | --- | --- | --- | --- | --- | --- | --- | --- |
|  | **AFP**  **group**  **(n=298)** | **AFP+US**  **group**  **(n=500)** | ***P* value** | **SMD** | **AFP**  **group**  **(n=280)** | **AFP+US**  **group**  **(n=280)** | ***P* value** | **SMD** | **AFP**  **group**  **(n=263)** | **AFP+US**  **group**  **(n=263)** | ***P* value** | **SMD** |
| Male sex | 200 (67.1%) | 360 (72.0%) | 0.168 | 0.106 | 195 (69.6%) | 188(67.1%) | 0.586 | 0.054 | 178 (67.7%) | 188 (71.5%) | 0.394 | 0.083 |
| Age (years) | 56 (50-64) | 56 (50-63) | 0.562 | 0.043 | 55 (50-63) | 57 (50-64) | 0.467 | 0.027 | 55 (51-63) | 56 (50-62) | 0.692 | 0.075 |
| Body mass index (kg/m^2^) | 24.6  (22.9-26.5) | 24.3  (22.3-26.1) | 0.116 | 0.114 | 24.6  (22.8-26.5) | 24.4  (22.6-26.2) | 0.860 | 0.019 | 24.5  (22.8-26.5) | 24.3  (22.3-26.2) | 0.486 | 0.062 |
| Alcohol consumption |  |  | 0.270 | 0.171 |  |  | 0.653 | 0.074 |  |  | 0.658 | 0.122 |
| None | 150 (50.3%) | 232 (46.4%) |  |  | 135 (48.2%) | 145 (51.8%) |  |  | 130 (49.4%) | 120 (45.6%) |  |  |
| Former | 109 (36.3%) | 182 (36.4%) |  |  | 106 (37.9%) | 96 (34.3%) |  |  | 95 (36.1%) | 100 (38.0%) |  |  |
| Current | 39 (13.1%) | 86 (17.2%) |  |  | 39 (13.9%) | 39 (13.9%) |  |  | 38 (14.4%) | 43 (16.3%) |  |  |
| Smoking habitus |  |  | 0.004 | 0.260 |  |  | 0.060 | 0.043 |  |  | 0.279 | 0.077 |
| None | 163 (54.7%) | 255 (51.0%) |  |  | 147 (52.5%) | 163 (58.2%) |  |  | 142 (54.0%) | 120 (45.6%) |  |  |
| Former | 104 (34.9%) | 149 (29.8%) |  |  | 103 (36.8%) | 77 (27.5%) |  |  | 91 (34.6%) | 100 (38.0%) |  |  |
| Current | 31 (10.4%) | 96 (19.2%) |  |  | 30 (10.7%) | 40 (14.3%) |  |  | 38 (14.4%) | 43 (16.3%) |  |  |
| Family history of HCC | 42 (14.1%) | 75 (15.0%) | 0.805 | 0.026 | 42 (15.0%) | 41 (14.6%) | >0.999 | 0.010 | 39 (14.8%) | 43 (16.3%) | 0.718 | 0.042 |
| Hypertension | 75 (25.2%) | 123 (24.6%) | 0.924 | 0.013 | 70 (25.0%) | 73 (26.1%) | 0.846 | 0.025 | 68 (25.9%) | 57 (21.7%) | 0.306 | 0.098 |
| Diabetes mellitus | 53 (17.8%) | 65 (13.0%) | 0.082 | 0.133 | 46 (16.4%) | 44 (15.7%) | 0.908 | 0.019 | 40 (15.2%) | 42 (16.0%) | 0.904 | 0.021 |
| HBV infection | 239 (80.2%) | 419 (83.8%) | 0.231 | 0.094 | 230 (82.1%) | 225 (80.4%) | 0.665 | 0.046 | 213 (81.0%) | 214 (81.4%) | >0.999 | 0.010 |
| HCV infection | 36 (12.1%) | 49 (9.8%) | 0.373 | 0.073 | 29 (10.4%) | 39 (13.9%) | 0.244 | 0.110 | 31 (11.8%) | 28 (10.6%) | 0.782 | 0.036 |
| Liver cirrhosis | 275 (92.3%) | 428 (85.6%) | 0.007 | 0.214 | 257 (91.8%) | 258 (92.1%) | >0.999 | 0.013 | 240 (91.3%) | 242 (92.0%) | 0.875 | 0.027 |
| Ascites | 13 (4.4%) | 11 (2.2%) | 0.130 | 0.122 | 10 (3.6%) | 10 (3.6%) | >0.999 | <0.001 | 6 (2.3%) | 9 (3.4%) | 0.600 | 0.069 |
| Platelet count (X10^3^/mm^3^) | 113 (83-151) | 134 (90-170) | <0.001 | 0.306 | 115 (85-152) | 116 (82-152) | 0.665 | 0.054 | 119 (86-154) | 116 (83-157) | 0.791 | 0.004 |
| MELD score | 8 (7-9) | 7 (7-9) | 0.074 | 0.133 | 8 (7-9) | 8 (7-9) | 0.909 | 0.009 | 8 (7-9) | 8 (7-9) | 0.681 | 0.028 |
| Infiltrative type of HCC |  |  |  |  | 14 ( 5.0%) | 14 ( 5.0%) | >0.999 | <0.001 | 12 (4.6%) | 13 (4.9%) | >0.999 | 0.189 |
| BCLC staging |  |  | <0.001 | 0.374 |  |  |  |  |  |  | 0.083 | 0.040 |
| Stage 0 | 94 (31.5%) | 82 (16.4%) |  |  | - | - | - |  | 78 (29.7%) | 62 (23.6%) |  |  |
| Stage A | 154 (51.7%) | 260 (52.0%) |  |  | - | - | - |  | 132 (52.1%) | 151 (57.4%) |  |  |
| Stage B | 19 (6.4%) | 88 (17.6%) |  |  | - | - | - |  | 18 (6.8%) | 29 (11.0%) |  |  |
| Stage C | 31 (10.4%) | 70 (14.0%) |  |  | - | - | - |  | 30 (11.4%) | 21 (8.0%) |  |  |
| Curative treatments | 4 (1.3%) | 282 (56.4%) | 0.347 | 0.074 | - | - | - |  | 108 (41.1%) | 100 (38.0%) | 0.532 | 0.062 |

* Matched by sex, age, body mass index, hepatitis B virus infection, hepatitis C virus infection, liver cirrhosis, ascites, platelet count, MELD score, and infiltrative type of HCC.

†Matched by BCLC stage, receipt of curative treatment, and all variables in Model 1.

AFP, alpha-fetoprotein; US, ultrasonography; SMD, standardized mean difference; HCC, hepatocellular carcinoma; HBV, hepatitis B virus; HCV, hepatitis C virus; MELD, model for end-stage liver disease; BCLC, Barcelona Clinic Liver Cancer.
