# Supplementary material for: Continued value of the serum alpha-fetoprotein test in surveilling at-risk populations for hepatocellular carcinoma
Source: PLoS One. 2020 Aug 26;15(8):e0238078. doi: 10.1371/journal.pone.0238078 (PMC7449471; doi:10.1371/journal.pone.0238078)
Supplement: S3 Table — (DOCX) [file pone.0238078.s007.docx]

**S3 Table.** Survival analyses in the AFP group with adjustment for possible lead time (based on the BCLC system)

|  | **Overall mortality** | | | | **Cancer-specific mortality** | | | |
| --- | --- | --- | --- | --- | --- | --- | --- | --- |
| Doubling time (days) | Median survival (years) | Unadjusted HR | Adjusted HR in Model 1 | Adjusted HR  in Model 2* | Median survival  (years) | Unadjusted HR | Adjusted HR  in Model 1 | Adjusted HR  in Model 2* |
| 90 | 2.86 | 0.75  (0.58-0.96) | 0.71  (0.55-0.92) | 0.82  (0.63-1.06) | 2.79 | 0.67  (0.49-0.90) | 0.64  (0.47-0.86) | 0.76  (0.56-1.03) |
| 120 | 2.71 | 0.77  (0.60-0.99) | 0.74  (0.57-0.95) | 0.87  (0.67-1.12) | 2.64 | 0.70  (0.52-0.94) | 0.67  (0.50-0.90) | 0.81  (0.60-1.10) |
| 150 | 2.57 | 0.79  (0.61-1.01) | 0.76  (0.59-0.98) | 0.89  (0.69-1.15) | 2.50 | 0.73  (0.54-0.98) | 0.70  (0.52-0.95) | 0.86  (0.63-1.17) |

*Adjusted for BCLC stage, receipt of curative treatment, and the variables in Model 1.

AFP, alpha-fetoprotein; BCLC, Barcelona clinic liver cancer; HR, hazard ratio.
