# Supplementary material for: Continued value of the serum alpha-fetoprotein test in surveilling at-risk populations for hepatocellular carcinoma
Source: PLoS One. 2020 Aug 26;15(8):e0238078. doi: 10.1371/journal.pone.0238078 (PMC7449471; doi:10.1371/journal.pone.0238078)
Supplement: S5 Table — (DOCX) [file pone.0238078.s009.docx]

**S5 Table.** Independent pre- and post-screening parameters related to survival in the cirrhotic subset (based on the BCLC system)

| **Variable** | **Overall morality** | | | | | | **Cancer-specific mortality** | | | | | |
| --- | --- | --- | --- | --- | --- | --- | --- | --- | --- | --- | --- | --- |
|  | **Model 1** | | | **Model 2*** | | | **Model 1** | | | **Model 2*** | | |
|  | **HR** | **95% CI** | ***P*** | **HR** | **95% CI** | ***P*** | **HR** | **95% CI** | ***P*** | **HR** | **95% CI** | ***P*** |
| **Group** |  |  |  |  |  |  |  |  |  |  |  |  |
| **AFP+US group** | 1 |  |  |  |  |  | 1 |  |  | 1 |  |  |
| **AFP group** | 0.59 | 0.45-0.77 | <0.001 | 0.67 | 0.51-0.87 | 0.003 | 0.52 | 0.37-0.71 | <0.001 | 0.59 | 0.43-0.82 | 0.002 |
| **US group** | 0.52 | 0.43-0.63 | <0.001 | 0.56 | 0.46-0.69 | <0.001 | 0.44 | 0.34-0.56 | <0.001 | 0.49 | 0.38-0.62 | <0.001 |
| **Male sex** | 1.20 | 0.93-1.54 | 0.170 | 1.13 | 0.87-1.46 | 0.366 | 1.70 | 1.26-2.28 | <0.001 | 1.57 | 1.17-2.11 | 0.002 |
| **Diabetes** | 1.29 | 1.04-1.59 | 0.019 | 1.19 | 0.96-1.46 | 0.110 | 1.31 | 1.01-1.69 | 0.042 | 1.17 | 0.91-1.52 | 0.228 |
| **Positive history of alcohol consumption** | 1.35 | 1.13-1.62 | 0.001 | 1.29 | 1.08-1.55 | 0.006 | 1.22 | 0.96-1.56 | 0.110 | 1.20 | 0.94-1.54 | 0.154 |
| **HBV infection** | 0.58 | 0.47-0.71 | <0.001 | 0.59 | 0.46-0.69 | <0.001 | 0.74 | 0.57-0.97 | 0.030 | 0.81 | 0.62-1.06 | 0.116 |
| **HCV infection** | 1.29 | 0.92-1.80 | 0.137 | 1.25 | 0.89-1.74 | 0.194 | - | - | - | - | - | - |
| **Ascites** | 2.31 | 1.56-3.42 | <0.001 | 2.06 | 1.38-3.08 | <0.001 | 2.78 | 1.69-4.57 | <0.001 | 2.36 | 1.43-3.90 | 0.001 |
| **MELD score** | 1.08 | 1.05-1.11 | <0.001 | 1.07 | 1.04-1.11 | <0.001 | 1.03 | 0.99-1.08 | 0.149 | 1.01 | 0.97-1.06 | 0.605 |
| **Platelet count <100k/mm^3^** | 1.39 | 1.15-1.67 | 0.001 | 1.16 | 0.96-1.41 | 0.120 | 1.27 | 1.01-1.69 | 0.042 | 0.97 | 0.76-1.23 | 0.796 |
| **Infiltrative type of HCC** | 5.54 | 4.01-7.67 | <0.001 | 2.18 | 1.51-3.15 | <0.001 | 6.61 | 4.65-9.40 | <0.001 | 2,14 | 1.44-3.18 | <0.001 |
| **BCLC stage** |  |  |  |  |  |  |  |  |  |  |  |  |
| **Stage 0** |  |  |  | 1 |  |  |  |  |  | 1 |  |  |
| **Stage A** |  |  |  | 1.43 | 1.09-1.87 | 0.009 |  |  |  | 1.84 | 1.27-2.68 | 0.001 |
| **Stage B** |  |  |  | 1.87 | 1.36-2.58 | <0.001 |  |  |  | 2.77 | 1.82-4.23 | <0.001 |
| **Stage C** |  |  |  | 4.59 | 3.27-6.44 | <0.001 |  |  |  | 8.33 | 5.41-12.82 | <0.001 |
| **Curative treatments** |  |  |  | 0.34 | 0.28-0.42 | <0.001 |  |  |  | 0.32 | 0.25-0.41 | <0.001 |

*****Adjusted for BCLC stage, receipt of curative treatment, and all variables in Model 1.

HCC, hepatocellular carcinoma; BCLC, Barcelona clinic liver cancer; HR, hazard ratio; CI, confidence interval; AFP, alpha-fetoprotein; US, ultrasonography; HBV, hepatitis B virus; HCV, hepatitis C virus; MELD, model for end-stage liver disease.
