# Supplementary material for: Continued value of the serum alpha-fetoprotein test in surveilling at-risk populations for hepatocellular carcinoma
Source: PLoS One. 2020 Aug 26;15(8):e0238078. doi: 10.1371/journal.pone.0238078 (PMC7449471; doi:10.1371/journal.pone.0238078)
Supplement: S6 Table — (DOCX) [file pone.0238078.s010.docx]

**S6 Table.** Survival analyses in the AFP group with adjustment for possible lead time in cirrhotic subset (based on the BCLC system)

|  | **Overall mortality** | | | | **Cancer-specific mortality** | | | |
| --- | --- | --- | --- | --- | --- | --- | --- | --- |
| Doubling time (days) | Median survival (years) | Unadjusted HR | Adjusted HR in Model 1 | Adjusted HR  in Model 2***** | Median survival  (years) | Unadjusted HR | Adjusted HR  in Model 1 | Adjusted HR  in Model 2***** |
| 90 | 3.06 | 0.71  (0.55-0.92) | 0.69  (0.53-0.90) | 0.81  (0.62-1.06) | 2.95 | 0.62  (0.45-0.85) | 0.61  (0.44-0.83) | 0.74  (0.54-1.03) |
| 120 | 3.02 | 0.74  (0.57-0.96) | 0.72  (0.55-0.94) | 0.86  (0.66-1.12) | 2.93 | 0.65  (0.47-0.89) | 0.63  (0.46-0.87) | 0.79  (0.57-1.09) |
| 150 | 2.92 | 0.75  (0.58-0.97) | 0.74  (0.57-0.96) | 0.88  (0.67-1.15) | 2.91 | 0.68  (0.49-0.93) | 0.66  (0.48-0.91) | 0.84  (0.61-1.16) |

*****Adjusted for BCLC stage, receipt of curative treatment, and the variables in Model 1.

AFP, alpha-fetoprotein; AJCC, American Joint Committee on Cancer; HR, hazard ratio
