# Supplementary material for: Continued value of the serum alpha-fetoprotein test in surveilling at-risk populations for hepatocellular carcinoma
Source: PLoS One. 2020 Aug 26;15(8):e0238078. doi: 10.1371/journal.pone.0238078 (PMC7449471; doi:10.1371/journal.pone.0238078)
Supplement: Attachment — Submitted filename: response letter_0723.docx [file pone.0238078.s011.docx]

**Continued Value of the Serum Alpha-Fetoprotein Test in Surveilling At-Risk Populations for Hepatocellular Carcinoma**

**Manuscript ID: PONE-D-20-16448**

Dear Editor,

We would like to thank the editors and reviewers for their careful consideration of our work. We believe that the suggestions made have strengthened our manuscript. A point-by-point response to the comments of the editors and reviewers follows.

============================================================

**Comments of Reviewer 1**

***#1.*** *I wonder who is the 10-15% of patients under surveillance without cirrhosis. It would be helpful for the reader to have a flowchart of case selection to figure out how you selected 1776 cases out of 9,615 patients*

**Reply:**

Most international guidelines advocate that, together with cirrhotic patients, non-cirrhotic patients with chronic hepatitis B, should undergo HCC surveillance, especially in the case of Asian populations (Heimbach JK et al., *Hepatology* 2018;67:358-380, Omata M et al., *Hepatol Int* 2017;11:317-370, Korean Liver Cancer Association and National Cancer Center, *Gut and Liver,* 2019;13:227-299) In line with these recommendations, most of the non-cirrhotic patients who were under surveillance were subjects with chronic hepatitis B (84.6%). In response to the reviewer’s comment, we have changed the flow chart of patient enrollment in Figure 1 as follows:

**Comments of Reviewer 2**

***#1.*** *AFP test and US are not therapeutic treatments. It does not make sense to compare with AFP+US and calculate survival rates. If patients are positive for both AFP and US, it is highly likely that tumors are big enough to be detected by both methods. Therefore, the survival rate of AFP+US is low. It does not mean that AFP test is useful to detect HCC or AFP can provide better survival rates or patients should take AFP tests. The authors mention that AFP surveillance could be helpful, but data in this study do not prove anything. The authors do not understand what they are doing.*

**Reply:**

We thank the reviewer for this helpful comment. First, as the reviewer mentioned, the AFP test and US are not therapeutic treatments but surveillance tools for detecting HCC, and the most important aim of surveillance is to reduce deaths from the target disease (Black WC et al., *J Natl Cancer Inst* 2002;94:167-73, Tabar L et al., *J Med Screen* 2002;9:159-62). Disease-specific mortality and all-cause mortality are used extensively as suitable end points in trials of cancer screening (Prasad V et al., BMJ 2016;352:h6080, Saquib N et al. *Int J Epidemiol* 2015;44:264-77). A randomized trial and several cohort studies have also demonstrated an association between HCC surveillance and early tumor detection and improved survival in patients with cirrhosis (Costentin CE et al., *Gastroenterology* 2018;155:431–442, Meer SV et al., *Journal of Hepatology* 2015;63:1156-1163, Zhang BH et al., *J Cancer Res Clin Oncol* 2004;130:417–422).

Second, as the reviewer suggested, the tumors detected by both AFP and US were significantly larger than those detected by AFP alone or US alone in our series. However, survival outcomes were not different between the AFP and AFP+US groups after multivariate analysis including tumor stage and anti-cancer treatment (i.e., model 2). These results were confirmed by a propensity score matching analysis, which has been added in response also to comment #2 of reviewer 2. This means that the prognoses of patients with HCC at the same stages would be similar regardless of the surveillance tests by which lesions were detected. Therefore, we speculated that AFP testing could play a worthwhile role in the early detection of HCC, complementing the limitations of US screening and improving survival by providing the opportunity for curative treatments.

***#2.*** *Demographic characteristics should be similar between groups. For example, AFP group has more female patients that other groups, and some studies have shown that female patients have better survival rates than male patients. Age, smoking, alcohol consumption are related to survival rates. Since NAFLD and obesity are associated with cancer development and metastases, BMI also influences survival rates. These parameters are significantly different between groups, and then how can the authors compare survival rates? This study is poorly designed and no data provided in this manuscript are useful for readers.*

**Reply:**

To overcome the retrospective design of our study in which demographic characteristics were not similar across the groups, we used a multivariate regression method including all relevant covariates related to the outcomes of interest. Furthermore, in response to the reviewer’s comment, we matched the AFP group with the AFP+US group for all confounding baseline variables in models 1 and 2 using a rigorous matching method based on propensity scores, and obtained similar results to the original non-matched analyses. We have added these findings in the Methods, Results and Discussion sections, Supplementary Table 2, and Supplementary Figures 2-3 as follows:

**(Methods) To reduce the impact of potential confounding effects in the AFP group and the AFP+US group, rigorous adjustment was made for significant differences in baseline characteristics in Model 1 and Model 2 by propensity score-based matching. Propensity scores were matched for the two groups based on differences of ±0.05 in the scores. Differences in overall mortality and cancer-specific mortality between the matched groups were compared using Cox regression models, with robust standard errors that accounted for the clustering of matched pairs.[24] A *P*<0.05 was considered statistically significant. All statistical analyses were performed using R package MatchIT.[25]**

**(Results)** **In addition, these results were recapitulated after PS matching between the AFP and AFP+US groups (S2 Table and S1-S2 Fig.).**

**(Discussion)** Because of the finding that this difference in survival between the patients identified by AFP with and without abnormal US findings was maintained after correcting for lead time, **combined with confounding adjustment**, we suggest that the observed survival benefit was due to downward stage migration followed by more frequent receipt of potentially curative treatment.

**S2 Table. Baseline characteristics of the AFP and AFP+US groups in the unmatched cohort, and propensity score-matched cohorts using variables included in Model 1 and Model 2, respectively**

| **Variable** | **Unmatched cohort** | | | | **Matched cohort (Model 1*)** | | | | **Matched cohort (Model 2†)** | | | |
| --- | --- | --- | --- | --- | --- | --- | --- | --- | --- | --- | --- | --- |
|  | **AFP**  **group**  **(n=298)** | **AFP+US**  **group**  **(n=500)** | ***P* value** | **SMD** | **AFP**  **group**  **(n=280)** | **AFP+US**  **group**  **(n=280)** | ***P* value** | **SMD** | **AFP**  **group**  **(n=263)** | **AFP+US**  **group**  **(n=263)** | ***P* value** | **SMD** |
| Male sex | 200 (67.1%) | 360 (72.0%) | 0.168 | 0.106 | 195 (69.6%) | 188(67.1%) | 0.586 | 0.054 | 178 (67.7%) | 188 (71.5%) | 0.394 | 0.083 |
| Age (years) | 56 (50-64) | 56 (50-63) | 0.562 | 0.043 | 55 (50-63) | 57 (50-64) | 0.467 | 0.027 | 55 (51-63) | 56 (50-62) | 0.692 | 0.075 |
| Body mass index (kg/m^2^) | 24.6  (22.9-26.5) | 24.3  (22.3-26.1) | 0.116 | 0.114 | 24.6  (22.8-26.5) | 24.4  (22.6-26.2) | 0.860 | 0.019 | 24.5  (22.8-26.5) | 24.3  (22.3-26.2) | 0.486 | 0.062 |
| Alcohol consumption |  |  | 0.270 | 0.171 |  |  | 0.653 | 0.074 |  |  | 0.658 | 0.122 |
| None | 150 (50.3%) | 232 (46.4%) |  |  | 135 (48.2%) | 145 (51.8%) |  |  | 130 (49.4%) | 120 (45.6%) |  |  |
| Former | 109 (36.3%) | 182 (36.4%) |  |  | 106 (37.9%) | 96 (34.3%) |  |  | 95 (36.1%) | 100 (38.0%) |  |  |
| Current | 39 (13.1%) | 86 (17.2%) |  |  | 39 (13.9%) | 39 (13.9%) |  |  | 38 (14.4%) | 43 (16.3%) |  |  |
| Smoking habitus |  |  | 0.004 | 0.260 |  |  | 0.060 | 0.043 |  |  | 0.279 | 0.077 |
| None | 163 (54.7%) | 255 (51.0%) |  |  | 147 (52.5%) | 163 (58.2%) |  |  | 142 (54.0%) | 120 (45.6%) |  |  |
| Former | 104 (34.9%) | 149 (29.8%) |  |  | 103 (36.8%) | 77 (27.5%) |  |  | 91 (34.6%) | 100 (38.0%) |  |  |
| Current | 31 (10.4%) | 96 (19.2%) |  |  | 30 (10.7%) | 40 (14.3%) |  |  | 38 (14.4%) | 43 (16.3%) |  |  |
| Family history of HCC | 42 (14.1%) | 75 (15.0%) | 0.805 | 0.026 | 42 (15.0%) | 41 (14.6%) | >0.999 | 0.010 | 39 (14.8%) | 43 (16.3%) | 0.718 | 0.042 |
| Hypertension | 75 (25.2%) | 123 (24.6%) | 0.924 | 0.013 | 70 (25.0%) | 73 (26.1%) | 0.846 | 0.025 | 68 (25.9%) | 57 (21.7%) | 0.306 | 0.098 |
| Diabetes mellitus | 53 (17.8%) | 65 (13.0%) | 0.082 | 0.133 | 46 (16.4%) | 44 (15.7%) | 0.908 | 0.019 | 40 (15.2%) | 42 (16.0%) | 0.904 | 0.021 |
| HBV infection | 239 (80.2%) | 419 (83.8%) | 0.231 | 0.094 | 230 (82.1%) | 225 (80.4%) | 0.665 | 0.046 | 213 (81.0%) | 214 (81.4%) | >0.999 | 0.010 |
| HCV infection | 36 (12.1%) | 49 (9.8%) | 0.373 | 0.073 | 29 (10.4%) | 39 (13.9%) | 0.244 | 0.110 | 31 (11.8%) | 28 (10.6%) | 0.782 | 0.036 |
| Liver cirrhosis | 275 (92.3%) | 428 (85.6%) | 0.007 | 0.214 | 257 (91.8%) | 258 (92.1%) | >0.999 | 0.013 | 240 (91.3%) | 242 (92.0%) | 0.875 | 0.027 |
| Ascites | 13 (4.4%) | 11 (2.2%) | 0.130 | 0.122 | 10 (3.6%) | 10 (3.6%) | >0.999 | <0.001 | 6 (2.3%) | 9 (3.4%) | 0.600 | 0.069 |
| Platelet count (X10^3^/mm^3^) | 113 (83-151) | 134 (90-170) | <0.001 | 0.306 | 115 (85-152) | 116 (82-152) | 0.665 | 0.054 | 119 (86-154) | 116 (83-157) | 0.791 | 0.004 |
| MELD score | 8 (7-9) | 7 (7-9) | 0.074 | 0.133 | 8 (7-9) | 8 (7-9) | 0.909 | 0.009 | 8 (7-9) | 8 (7-9) | 0.681 | 0.028 |
| Infiltrative type of HCC |  |  |  |  | 14 ( 5.0%) | 14 ( 5.0%) | >0.999 | <0.001 | 12 (4.6%) | 13 (4.9%) | >0.999 | 0.189 |
| BCLC staging |  |  | <0.001 | 0.374 |  |  |  |  |  |  | 0.083 | 0.040 |
| Stage 0 | 94 (31.5%) | 82 (16.4%) |  |  | - | - | - |  | 78 (29.7%) | 62 (23.6%) |  |  |
| Stage A | 154 (51.7%) | 260 (52.0%) |  |  | - | - | - |  | 132 (52.1%) | 151 (57.4%) |  |  |
| Stage B | 19 (6.4%) | 88 (17.6%) |  |  | - | - | - |  | 18 (6.8%) | 29 (11.0%) |  |  |
| Stage C | 31 (10.4%) | 70 (14.0%) |  |  | - | - | - |  | 30 (11.4%) | 21 (8.0%) |  |  |
| Curative treatments | 4 (1.3%) | 282 (56.4%) | 0.347 | 0.074 | - | - | - |  | 108 (41.1%) | 100 (38.0%) | 0.532 | 0.062 |

* Matched by sex, age, body mass index, hepatitis B virus infection, hepatitis C virus infection, liver cirrhosis, ascites, platelet count, MELD score, and infiltrative type of HCC.

†Matched by BCLC stage, receipt of curative treatment, and all variables in Model 1.

AFP, alpha-fetoprotein; US, ultrasonography; SMD, standardized mean difference; HCC, hepatocellular carcinoma; HBV, hepatitis B virus; HCV, hepatitis C virus; MELD, model for end-stage liver disease; BCLC, Barcelona Clinic Liver Cancer.

**S1 Fig. (A) overall mortality and (B) cancer-specific mortality of the AFP and AFP+US groups after propensity score matching in Model 1. Both overall and cancer-specific mortalities were significantly different in the two groups (Ps<0.001, respectively, by the log-rank test)**

**S2 Fig. (A) overall mortality and (B) cancer-specific mortality of the AFP and AFP+US groups after propensity score matching in Model 2. Neither overall nor cancer-specific mortalities were significantly different between the two groups (P=0.200 and P=0.230, respectively by the log-rank test).**

**Comments of Reviewer 3**

***#1.*** *Since the study focuses on the continued value of AFP, it’s better to explain more about its original value in HCC screening. For example, in the part of introduction, the function of AFP and ultrasound screening should be explained more clearly. What are their advantages and disadvantages respectively? Moreover, what about their combinations?*

**Reply:**

Thank you for your helpful comment. We have added the more relevant content to the Introduction section of the revised manuscript as follows:

(**2^nd^ paragraph of Introduction**) Surveillance for HCC is thought to be a way to detect lesions at an earlier stage and improve clinical outcomes in asymptomatic at-risk populations.[4, 5] Currently, ultrasonography (US) is regarded as the backbone of screening for HCC, but it has practical limitations in terms of high operator-dependency and variable sensitivity. **A recent meta-analysis highlights the suboptimal (<50%) sensitivity of US for detection of HCC at an early stage, although the sensitivity and specificity of US in detecting HCC of any stage exceed 90% (Tzartzeva K et al., *Gastroenterology* 2018;154:1706–1718).** The serum alpha-fetoprotein (AFP) assay is the only serological screening tool for early detection of HCC that reliably meets the final 5-phases criterion for possibly reducing the population disease burden listed by the Early Detection Research Network (EDRN), an initiative of the National Cancer Institute (NCI).[6] However, the use of AFP in surveillance is subject to ongoing debate, even as an adjunct to USG, due to issues about cost-effectiveness.[7-9] **There have been false-positive results for HCC detection due to the AFP elevation encountered in chronic liver disease, and false-negative results in HCCs not secreting AFP (Wong RJ et al*., Clin Liver Dis* 2015;19:309–323).** Unfortunately, there are no robust or promising next-generation biomarkers available for clinical use in screening or diagnostic systems

**Both the** **American Association for the Study of Liver Diseases (AASLD) and the National Comprehensive Cancer Network (NCCN) guidelines for HCC, newly updated in 2017, re-introduce serum AFP testing as an additional surveillance method because of its potential benefit for patients with HCCs secreting AFP (Heimbach JK et al., *Hepatology* 2018;67:358-380, Benson AB el al, *J Natl Compr Canc Netw 2017*;15:563-573). Recent interesting efforts in the U.S. to develop a computer model for detecting HCC also included serum AFP values in the prediction calculator (El-Serag HB, *Gastroenterology* 2014;146:1249-1255, White DL, *Gastroenterology* 2015;149:1986-1987).**

***#2.*** *There is something not very clear for the patient enrollment. It’s written that the study population consists of 9,615 patients, among which 5,786 are excluded because they did not undergo biannual regular surveillance tests for HCC. What kind of population are required to participate in this surveillance program? Are there any criteria?*

**Reply:**

The practice guidelines of the Korean Liver Cancer Society for the management of HCC recommend that surveillance for HCC should be performed with liver US plus serum AFP measurement every 6 months in high-risk subjects (i.e., patients with chronic hepatitis B, chronic hepatitis C, and liver cirrhosis) (Korean Liver Cancer Association and National Cancer Center, *Gut and Liver*, 2019;13:227-299). However, the HCC surveillance rate in real-world practice is quite low. A Korean tertiary hospital cohort study found that the previous surveillance rate was only 39.8% in newly diagnosed HCC patients, and adherence to the strict 6-monthly surveillance rate of the current guidelines was only 21.9%, suggesting a suboptimal surveillance status (Im S et al., *Cancer Res Treat* 2019;51:1357-1369). Routine surveillance also occurred in only 12.0% of the HCV-infected population, based on U.S. Veterans Health Administration data (Davila JA et al., *Ann Intern Med.* 2011;154:85–93) Since the objective of the study was to evaluate the effect of surveillance of serum AFP on a background of US examination, we specifically included asymptomatic patients who underwent standard surveillance for HCC at 6-monthly intervals.

**Journal Requirements:**

*#1. When submitting your revision, we need you to address these additional requirements. Please ensure that your manuscript meets PLOS ONE's style requirements, including those for file naming.*

**Reply:**

We have checked that our manuscript meets PLOS ONE’s style requirements.

*#2. To comply with PLOS ONE submission guidelines, in your Methods section, please provide additional information regarding your statistical analyses. For more information on PLOS ONE's expectations for statistical reporting, please see* [*https://journals.plos.org/plosone/s/submission-guidelines.#loc-statistical-reporting*](https://journals.plos.org/plosone/s/submission-guidelines.#loc-statistical-reporting)*.*

**Reply:**

We have moved our statistical analyses into the Methods section.

*#3. Please include your tables as part of your main manuscript and remove the individual files. Please note that supplementary tables (should remain/ be uploaded) as separate "supporting information" files*

**Reply:** We have included tables as part of the main manuscript and removed the individual files

*#4. Please include captions for your Supporting Information files at the end of your manuscript, and update any in-text citations to match accordingly.*

**Reply:** We have included captions for our Supporting Information files at the end of our manuscript.
